# Reassessing the Chromosome Number and Morphology of the Turtle Ant *Cephalotes pusillus* (Klug, 1824) Using Karyomorphometrical Analysis and Observations of New Nesting Behavior

**DOI:** 10.3390/insects8040114

**Published:** 2017-10-23

**Authors:** Maykon Passos Cristiano, Tássia Tatiane Pontes Pereira, Laysa Peneda Simões, Vivian Eliana Sandoval-Gómez, Danon Clemes Cardoso

**Affiliations:** 1 Departamento de Biodiversidade, Evolução e Meio Ambiente/ICEB, Universidade Federal de Ouro Preto, Campus Morro do Cruzeiro, Ouro Preto, Minas Gerais 35400-000, Brazil; maykoncristiano@hotmail.com; 2 Programa de Pós-graduação em Ecologia, Universidade Federal de Viçosa, Viçosa, Minas Gerais 36570-000, Brazil; tassia.pontes@gmail.com; 3 Programa de Pós-graduação em Biotecnologia, Universidade Federal de Ouro Preto, Campus Morro do Cruzeiro, Ouro Preto, Minas Gerais 35400-000, Brazil; laysapenedas@gmail.com; 4 161 Ashridge Road, Darra, Queensland 4076, Australia; vivian.sandoval@gmail.com

**Keywords:** *Cephalotes*, Cytogenetics, Formicidae, nesting, karyotype, Karyomorphometry

## Abstract

Here we use karyomorphometrical analysis to characterize and evaluate the karyotype of the turtle ant *Cephalotes pusillus* (Klug, 1824). This is the first representative of this diverse ant genus to be cytogenetically studied. They bear a diploid chromosome set of 44 chromosomes, which, according to the centromeric index, are metacentric, submetacentric, and subtelocentric. This small ant is quite widely distributed in the Neotropics and seems to be well adapted to living in disturbed areas. Here we report the species nesting on dead trunks used to build fences at countryside houses and farms. On these nests, we observed some never reported behavior of *C. pusillus*: the ants appear to be able to dig by actively removing small fragments of dead wood fiber, hence expanding their nest cavities. It was not thought that *Cephalotes* species had this ability, given that they nest in preexisting cavities. Our observations are initial remarks that the small plier-like mandibles of *C. pusillus* may not be a constraint for this species, adding to our knowledge on ant nesting biology.

## 1. Introduction

Myrmicinae is the most diverse ant subfamily, comprising 142 genera and about 6650 valid species, inhabiting almost all biogeographic regions of the world [1,2]. *Cephalotes* Latreille, 1802 is a genus of arboreal myrmicine ants, commonly known as turtle ants due to their shell-like armor, that nest in pre-existing vegetal cavities and possess the most specialized insect proventriculus [3,4,5]. This genus contains 135 species, with 116 species limited to the Neotropics and three reaching the Nearctic region in the south of Florida, Texas, and Arizona, of which 16 are fossils from Dominican and Mexican amber [2,3]. In the New World, *Cephalotes* is the third most diverse ant genus after *Camponotus* Mayr, 1861 and *Pheidole* Westwood, 1839 [3].

Phylogenetic analyses of *Cephalotes* confirm the monophyly of this genus and its position as the sister group of *Procryptocerus* Emery, 1887 based on morphologic [3] and molecular data [5,6,7]. Currently, the *Cephalotes* and *Procryptocerus* genera are considered to be the sister clade of *Pheidole*, within the tribe Attini [1]. Karyotype features of ants are virtually unavailable, regardless of the number of species that have been cytogenetically studied, which is not more than 800 [8]. The majority of these studies only report the chromosome number and the morphology of the chromosomes, without any karyomorphometrical analysis. The genus *Cephalotes* is not an exception and until now nothing has been presented in the literature about the karyotypes of the species therein, except for a conference abstract from 14 years ago [9].

In order to contribute to the cytogenetic knowledge of Formicidae and increase our understanding of karyotype evolution in this important group of insects, the present study aimed to characterize the karyotype of *Cephalotes pusillus* (Klug, 1824) with chromosome measurements. When sampling for *C. pusillus* colonies for cytogenetic analyses, we surprisingly found colonies living in dead trunks used to build fences. Searching in the literature, we did not find any report of such behavior, including from the digging behavior by *C. pusillus* workers. Indeed, we found contradictory statements with respect to this. Thus, we also report these new observations of *C. pusillus*, nesting, and digging behavior, based on three colonies.

## 2. Material and Methods

Two colonies of *Cephalotes pusillus* were sampled from nests in dead tree trunks that are used to build fences in farms and countryside houses. The first colony was sampled in Viçosa (20°43′09.7″ S 42°50′50.9″ W) and another colony was sampled in Ouro Preto (20°21′53.4″ S 43°38′21.9″ W), both in Minas Gerais State, Brazil. The whole trunk was transported to the laboratory and workers, queens, and broods (pupae and larvae) were collected. All individuals were transferred to plastic containers and kept in the laboratory [10]. A third colony was only observed and recorded in Belém (1°28′04.3″ S 48°26′56.6″ W), Pará State.

We obtained larvae from the two colonies from Minas Gerais State to perform karyotype characterization. Ten individuals from each colony were used in cytogenetic analyses. Metaphase spreads were prepared from the cerebral ganglia of post-defecant larvae, according to the protocol proposed by Imai et al. [11]. The cerebral ganglion was dissected in colchicine-hypotonic solution (0.005%) under a stereoscopic microscope. Next, the ganglion was transposed to a new drop of colchicine-hypotonic solution and incubated under light protection for one hour until slide preparation (for detailed descriptions of the procedure see [11,12]). All slides with metaphases were stained with 4% Giemsa solution in Sorensen’s buffer, pH 6.8.

The best metaphases were photographed using an Olympus BX40 and BX51 microscope (Olympus Corporation, Tokyo, Japan) equipped with a camera, Olympus^®^ DP72. The brightness and contrast of the karyotypes were optimized and the karyotypes were analyzed in image-editing software. The chromosome morphology was evaluated using a karyomorphometrical approach. We observed 10–15 metaphases per slide for each individual worker. The chromosomal morphology was determined based on the arm ratio [13]. The chromosomes were classified as metacentric (M), submetacentric (SM), subtelocentric (ST), and telocentric (T). We measured eight good spread metaphases with chromosomal integrity and evident centromeres, and without overlapping during the chromosome measurements. The metaphases were measured by using Image-Pro Plus software (Media Cybernetics, LP, USA) and values calibrated by the scale bar and transferred to Excel^®^ (Microsoft, Redmond, WA, USA). Each metaphase was measured by crossing a line along each individual chromosome from end point to the centromere representing long arm length (L) and short arm length (S). Then, the arms of each chromosome were summed in order to obtain the total length (TL), and a second measurement of TL was carried by crossing a line end-to-end of the chromosome. The results of each metaphase were averaged. The following other features of chromosomes were evaluated: arm ratio between the long and short arms (*r* = L/S), relative chromosome length (RL) of each chromosome (TL × 100/∑TL), and asymmetric index (∑long arms/∑total length × 100). The arm ratio, *r*, for metacentric chromosomes should be 1.0–1.7, for submetacentric 1.7–3.0, and for subtelocentric 3.0–7.0 and telocentric values above 7.0 [13]. In order to evaluate the degree of variation and validate our measured karyotype, we calculate the coefficient of variation (CV) of karyotype length for each specimen measured. The karyotype length (KL) was obtained as a sum of the TL of each chromosome complement and averaged by the diploid set of chromosomes (2n = 44), resulting in the mean KL. Then, the CV was calculated dividing the standard deviation by the mean KL. We also carried out generalized linear models (GLM) analyses to check differences of CVs and mean KLs among specimens.

Before colony collection for cytogenetic analysis, we also observed the two nests of *Cephalotes pusillus* in the fences of countryside houses and farms in the municipality of Viçosa, Minas Gerais State, Brazil, for 28 days from July to August 2013. Every morning between 7 a.m. and 9 a.m. we observed polydomous colonies (ant colonies that occupy multiple nests at the same time) for ten minutes in order to record the maintenance of digging behavior. Additionally, we have observed the behavior on an additional colony from Belém, Pará State. In the interest of documenting the digging behavior we have recorded some small videos with help of a digital camera summarizing 5 min and 3 s. On the base of these videos we have established an ethogram.

## 3. Results and Discussion

### 3.1. Cephalotes pusillus Karyotype Analysis

**Figure 1:**
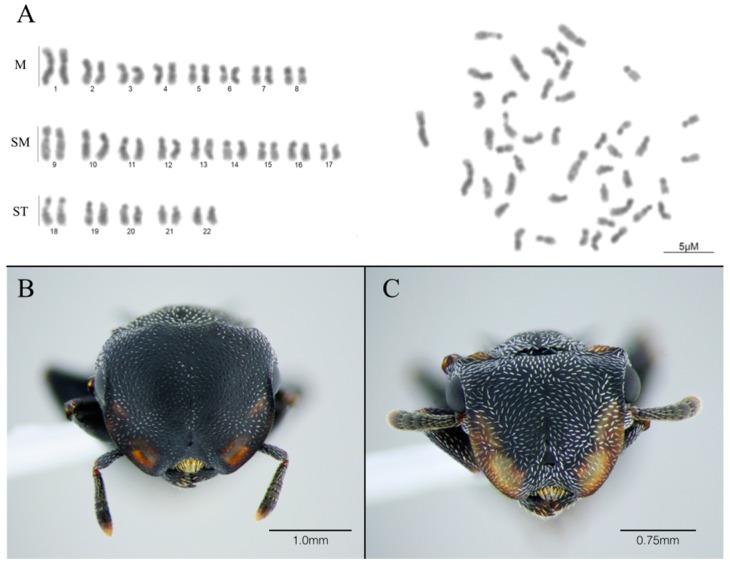
Conventional staining of the complete chromosome set of *Cephalotes pusillus*. Karyotype and metaphase of female workers of *C. pussilus* 2n = 44 sorted according to centromeric index (**A**). Automontage front-view pictures of *C. pussilus* soldier (**B**) and worker (**C**).

The chromosome number observed for both *C. pusillus* colonies was 2n = 44 (Figure 1), which is in agreement with previous chromosome counts for this species available in a conference abstract [9]. The karyomorphometrical analysis revealed that the karyotype of this species consists of eight metacentric (M) pairs, nine submetacentric (SM) pairs, and five subtelocentric (ST) pairs. Considering this chromosome classification, the karyotype formula for the diploid set would be 2K = 16M + 18SM + 10ST. However, the only previous report for chromosome number in the *Cephalotes* genus reported that the *C. pusillus* karyotype shows nine acrocentric, rather than submetacentric or subtelocentric, pairs, as denoted in the present study. In this case, the karyotype formula would be 2K = 28M + 16A [9]. The authors of the previous karyotype report did not specify how the chromosome morphology was determined. The average long-arm measurements found in this study ranged from 1.07 ± 0.19 to 2.20 ± 0.32 μM, and average short-arm measurements ranged from 0.41 ± 0.08 μM to 1.44 ± 0.37 μM. This results in arm ratios between 1.29± 0.15 μM and 3.66 ± 0.51μm (Table 1), which support our finding that the karyotype is composed of metacentric, submetacentric, and subtelocentric chromosomes, but not acrocentric [13].

Chromosome lengths vary from 3.48 ± 0.7 μM to 1.78 ± 0.34 μM for the species, which characterize a karyotype with medium to small chromosomes [14], since medium to large would be expected for chromosome lengths higher than 4 μM. Since studies on chromosome measurements in ants are scarce, general conclusions about chromosome size in ants are still unfeasible. *Acromyrmex striatus* is one species whose chromosome measurements are available [15]. It presents a diploid chromosome number of 22, and shows larger metacentric and submetacentric chromosomes. The relationship between chromosome number and chromosome length will be possible to test for ants when much more data become available.

**Figure 2:**
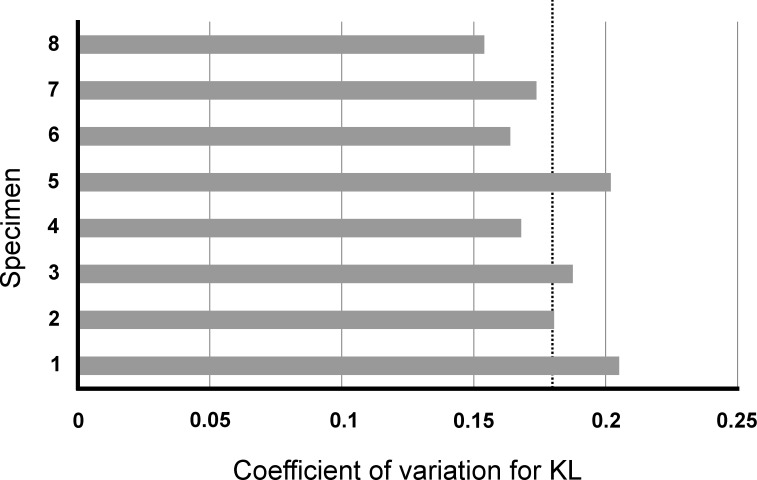
Coefficient of variation (CV) of karyotype length. CVs for karyotype length of the specimens of *Cephalotes pusillus* measured. The dash line represents the mean CV.

Coefficient of variation was calculated for the total length of karyotypes as well as the mean karyotype of each specimen measured (Supplement Table S1). The CVs of each specimen are plotted in Figure 2. All measurements had low variability (i.e., less than one fold standard deviation from the average CV). Further, the CVs were not significantly different (GLM: *df* = 1, deviance = 0.0010, *p* = 0.06), as well as the mean KLs (GLM: *df* = 1, deviance = 0.2317, *p* = 0.18). All these results together indicate that the averaged measurements of chromosomes represent a good and stable feature of *C. pusillus* karyotype. Further, this study provides a baseline for karyomorphometrical measurements that may be used to evaluate trends on the evolution of ants’ genome.

The total length of all chromosomes and the asymmetric index are 100.51 µm and 65.71 µM, respectively. This index, together with the presence of majority of small to medium metacentric and submetacentric chromosomes, suggests a tendency towards a symmetrical karyotype. This symmetry in chromosome morphology could be accomplished by increasing heterochromatin after chromosome fission events, as suggested in *Mycetophylax simplex* [16], which presents only metacentric and submetacentric chromosomes. However, chromosome paracentromeric inversion cannot be rejected as a possible way of forming such a chromosome set configuration. These results do not represent the absence of chromosome rearrangements and stability in symmetric karyotypes. Further banding analysis and characterization of other species would be necessary to provide a better framework for understanding chromosome changes in *Cephalotes*.

### 3.2. Cephalotes pusillus Nesting and Digging Behavior

**Figure 3:**
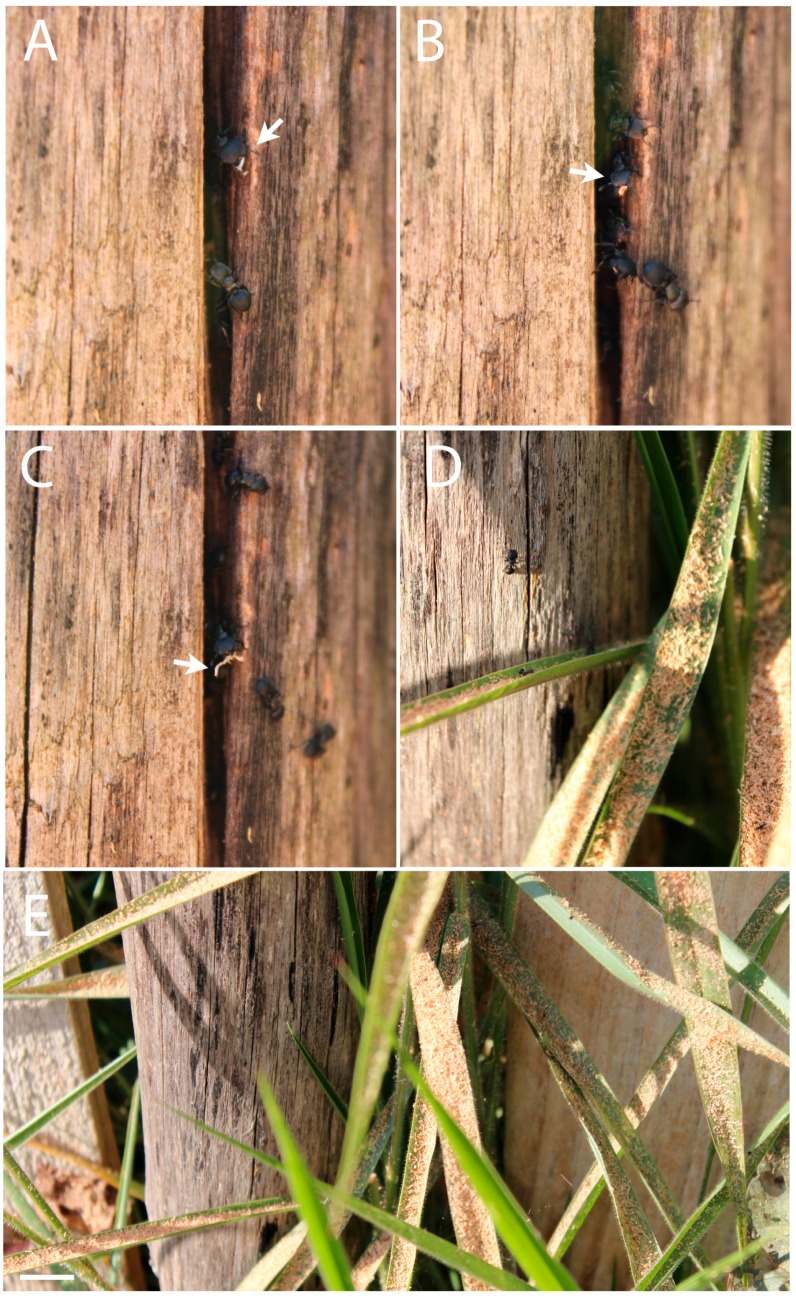
*Cephalotes pusillus* digging behavior records. (**A**–**C**) Workers unloading the small fragments of wood or sawdust; (**D**,**E**) accumulation of sawdust thrown away by the workers above the vegetation under nest.

Few authors have reported the nesting biology of *Cephalotes* species and detailed descriptions are scarce. De Andrade and Baroni-Urbani [3] performed the most comprehensive study on *Cephalotes* and recently much more knowledge about its ecology and evolution has been accumulated [4,5]. It is a consensus that *Cephalotes* species inhabit pre-existing cavities inside dead wood or in broken ends of dead branches, or in dead or live trees [3,4,5,17]. In general, these pre-existing cavities were made and abandoned by insects, and the ants appeared to not be able to modify or enlarge them to a great extent [4]. Astonishingly, we observed *C. pusillus* nesting in dead trunks of trees that had been used to build fences in countryside houses and farms in Brazil (Figure 3). This may represent a new nesting opportunity for this ant, which is much smaller than *Cephalotes atratus* (Linnaeus, 1758) and occurs sympatrically with this species but has never been observed in such fence nests. This behavior was also observed in other sites in Brazil (Tocantins and Pará States) and Colombia (V.E. Sandoval-Gómez personal observations). This nesting behavior seems to be common for this species and has not, to our knowledge, so far been reported in the literature. 

A study from Del-Claro et al. [18] has reported a detailed ethogram of soldiers and workers of *C. pusillus* from colonies sampled in Cerrado. The main important findings of this study were that *C. pusillus* is diurnal and that the main food source was extrafloral nectar and honeydew from homopterans, and that the majority of the individuals stayed inside the colony resting [18]. Based on our video records we have observed five categories displayed by workers and soldiers: “antennation”, “patrol”, “sawdust removing”, “exploring”, and “grooming”. Since we could not differentiate among individuals, only the behavior category was measured. When workers and soldiers go out of the colony and after go inside the colony, we count this as “exploring”. We observed this 134 times, whereas 12 times they went out to “patrol”, running or stopping around the nest entrance. Grooming was observed 11 times followed by 58 antennations. In the interval evaluated (five min. recorded) workers have been observed removing sawdust 86 times.

**Figure 4:**
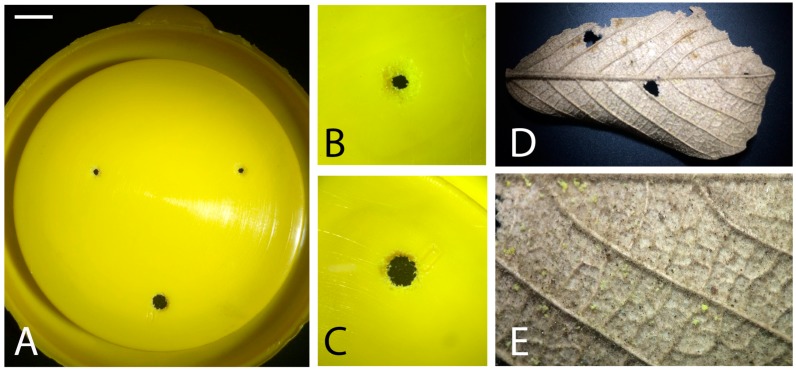
Laboratory observations of *Cephalotes pusillus* digging behavior. The figure shows holes and plastic fragments from lids of rearing systems. (**A**–**C**) The workers were able to make the small holes bigger to escape. (**D**,**E**) Plastic yellow fragments on leaf.

*Cephalotes pusillus* appears to be taking advantage of the space left by the death of the woody element in the stem to establish its colony in fences. Numerous trunks are used to build variable lengths of fencing, and the ants can then spread their nests along several trunks into polydomous colonies. The wires that connect the fences’ trunks serve as trails for workers that travel from one nest to the other. Polydomy allows *Cephalotes* species to meet the needs of a large colony without being limited by the size of the nest cavity occupied [4,17]. Although it has been proposed that *Cephalotes* species are not able to greatly expand the nest cavities, we observed a daily accumulation of sawdust above the nest (Figure 3). Every morning numerous workers were observed going inside the trunk cavities and coming back to the nest entrance to unload sawdust (Supplement Video S2). Powell [4] reported *Cephalotes* workers removing frass left by a wood-boring insect, the original occupant of the trunk, but not removing sawdust. The amount and irregularity of sawdust fragments suggests that the *C. pusillus* workers were removing the dead wood pieces to better accommodate the colony. It seems that this species has an ability to dig in quite hard wood, despite their small plier-like mandibles [3,4], or that the quality and hardness of the wood used in this case to construct the fence (*Eucalyptus grandis*, W. Hill ex Maiden) was an advantage to this colony of *C. pusillus*. We also observed during nest rearing under laboratory conditions that *C. pusillus* was able to expand small holes in the system used to keep the colonies in laboratory conditions (Figure 4). They were able to create bigger holes by “digging” the plastic yellow lid in all three colonies maintained in our laboratory (Figure 4). Further analysis should be performed in order to improve our understanding of the nesting abilities of *C. pusillus,* as well other *Cephalotes* species.

## 4. Conclusions

The analysis shows that *Cephalotes pusillus* has a diploid number of 44 chromosomes. The karyotype consists of eight metacentric pairs, nine submetacentric pairs, and five subtelocentric pairs, which represent a karyotype formula 2K = 16M + 18SM + 10ST. The karyomorphometrical analysis revealed that the averaged measurements of chromosomes represent a good and stable feature of the *C. pusillus* karyotype. Therefore, our analysis provides a baseline for karyomorphometrical measurements that may be used to evaluate trends on genome evolution. The colonies cytogenetically studied of *Cephalotes pusillus* were sampled on dead trunks that are used to build fences. It seems that altered countryside environments may not impact *C. pusillus* nesting and dispersion. Besides, the workers appear to be able to dig by actively removing small fragments of dead wood fiber, hence expanding their nest cavities.

## Figures and Tables

**Table 1:** Karymorphometrical analyses of *Cephalotes pusilus* (Klug, 1824). TL: total length; L: long arm length; S: short arm length; RL: relative length; *r*: arm ratio (= L/S).

Chromosome	TL (µM)	L (µM)	S (µM)	RL	*r*	Chromosome Classification
1	3.48 ± 0.7	1.94 ± 0.39	1.44 ± 0.37	3.45 ± 0.29	1.37 ± 0.14	Metacentric
1	3.26 ± 0.74	1.87 ± 0.41	1.38 ± 0.33	3.22 ± 0.33	1.37 ± 0.12	Metacentric
2	2.62 ± 0.45	1.47 ± 0.28	1.12 ± 0.18	2.6 ± 0.13	1.31 ± 0.12	Metacentric
2	2.54 ± 0.43	1.44 ± 0.25	1.11 ± 0.22	2.52 ± 0.11	1.3 ± 0.12	Metacentric
3	2.4 ± 0.41	1.31 ± 0.25	0.98 ± 0.17	2.38 ± 0.14	1.35 ± 0.23	Metacentric
3	2.3 ± 0.38	1.33 ± 0.21	0.92 ± 0.15	2.28± 0.07	1.46 ± 0.13	Metacentric
4	2.25 ± 0.35	1.33 ± 0.25	0.92 ± 0.13	2.24 ± 0.06	1.45 ± 0.17	Metacentric
4	2.19 ± 0.33	1.26 ± 0.22	0.89 ± 0.1	2.18 ± 0.06	1.41 ± 0.16	Metacentric
5	2.14 ± 0.32	1.26 ± 0.21	0.91 ± 0.17	2.13 ± 0.06	1.4 ± 0.17	Metacentric
5	2.09 ± 0.31	1.17 ± 0.18	0.91 ± 0.14	2.09 ± 0.08	1.29 ± 0.15	Metacentric
6	2.04 ± 0.31	1.21 ± 0.22	0.86 ± 0.13	2.03 ± 0.06	1.41 ± 0.21	Metacentric
6	2.02 ± 0.32	1.18 ± 0.23	0.83 ± 0.09	2.01 ± 0.08	1.41 ± 0.18	Metacentric
7	1.99 ± 0.32	1.19 ± 0.21	0.8 ± 0.11	1.98 ± 0.07	1.48 ± 0.15	Metacentric
7	1.92 ± 0.33	1.08 ± 0.18	0.81 ± 0.09	1.9 ± 0.07	1.33 ± 0.17	Metacentric
8	1.85 ± 0.32	1.18 ± 0.24	0.82 ± 0.15	1.84 ± 0.08	1.45 ± 0.15	Metacentric
8	1.8 ± 0.33	1.07 ± 0.19	0.79 ± 0.13	1.78 ± 0.09	1.37 ± 0.16	Metacentric
9	3.27 ± 0.42	2.2 ± 0.32	1.01 ± 0.12	3.27 ± 0.11	2.2 ± 0.25	Submetacentric
9	3.09 ± 0.51	2.05 ± 0.33	1.03 ± 0.21	3.07 ± 0.14	2.03 ± 0.35	Submetacentric
10	2.8 ± 0.39	1.92 ± 0.25	0.84 ± 0.18	2.79 ± 0.1	2.34 ± 0.39	Submetacentric
10	2.66 ± 0.42	1.72 ± 0.33	0.86 ± 0.2	2.65 ± 0.11	2.01 ± 0.27	Submetacentric
11	2.54 ± 0.33	1.69 ± 0.22	0.81 ± 0.13	2.53 ± 0.12	2.13 ± 0.3	Submetacentric
11	2.47 ± 0.3	1.69 ± 0.24	0.76 ± 0.12	2.47 ± 0.12	2.25 ± 0.28	Submetacentric
12	2.4 ± 0.3	1.63 ± 0.25	0.74 ± 0.12	2.4 ± 0.1	2.21 ± 0.32	Submetacentric
12	2.36 ± 0.3	1.56 ± 0.23	0.76 ± 0.1	2.35 ± 0.1	2.05 ± 0.26	Submetacentric
13	2.31 ± 0.31	1.53 ± 0.23	0.75 ± 0.09	2.3 ± 0.08	2.06 ± 0.25	Submetacentric
13	2.28 ± 0.31	1.52 ± 0.18	0.73 ± 0.15	2.27 ± 0.06	2.11 ± 0.23	Submetacentric
14	2.21 ± 0.32	1.53 ± 0.26	0.68 ± 0.08	2.21 ± 0.05	2.28 ± 0.36	Submetacentric
14	2.17 ± 0.32	1.46 ± 0.26	0.66 ± 0.1	2.16 ± 0.05	2.25 ± 0.41	Submetacentric
15	2.13 ± 0.34	1.36 ± 0.25	0.72 ± 0.09	2.12 ± 0.08	1.89 ± 0.21	Submetacentric
15	2.09 ± 0.32	1.38 ± 0.23	0.65 ± 0.13	2.08 ± 0.09	2.16 ± 0.34	Submetacentric
16	2.06 ± 0.33	1.4 ± 0.23	0.66 ± 0.09	2.05 ± 0.11	2.14 ± 0.29	Submetacentric
16	2.01 ± 0.33	1.3 ± 0.37	0.75 ± 0.28	2 ± 0.13	1.92 ± 0.68	Submetacentric
17	1.94 ± 0.27	1.32 ± 0.17	0.61 ± 0.11	1.94 ± 0.13	2.19 ± 0.34	Submetacentric
17	1.81 ± 0.3	1.21 ± 0.22	0.58 ± 0.08	1.8 ± 0.17	2.09 ± 0.28	Submetacentric
18	2.42 ± 0.38	1.91 ± 0.32	0.52 ± 0.06	2.41 ± 0.09	3.66 ± 0.51	Subtelocentric
18	2.33 ± 0.36	1.8 ± 0.32	0.54 ± 0.1	2.32 ± 0.07	3.36 ± 0.37	Subtelocentric
19	2.24 ± 0.32	1.7 ± 0.24	0.54 ± 0.11	2.23 ± 0.08	3.22 ± 0.39	Subtelocentric
19	2.18 ± 0.31	1.7 ± 0.24	0.48 ± 0.06	2.17 ± 0.06	3.59 ± 0.46	Subtelocentric
20	2.13 ± 0.28	1.59 ± 0.24	0.5 ± 0.08	2.12 ± 0.08	3.21 ± 0.58	Subtelocentric
20	2.09 ± 0.3	1.61 ± 0.22	0.5 ± 0.08	2.08 ± 0.05	3.25 ± 0.22	Subtelocentric
21	2.01 ± 0.33	1.52 ± 0.19	0.5 ± 0.08	2 ± 0.08	3.1 ± 0.41	Subtelocentric
21	1.95 ± 0.32	1.54 ± 0.26	0.45 ± 0.06	1.94 ± 0.07	3.44 ± 0.58	Subtelocentric
22	1.89 ± 0.34	1.47 ± 0.24	0.47 ± 0.09	1.88 ± 0.12	3.14 ± 0.31	Subtelocentric
22	1.78 ± 0.34	1.45 ± 0.27	0.41 ± 0.08	1.76 ± 0.12	3.54 ± 0.55	Subtelocentric
∑	100.51					

## Supplementary Materials

The following are available online at www.mdpi.com/2075-4450/8/4/114/s1, Table S1: Karyomorphometrical analyses of the eight specimens of *Cephalotes pusillus* (Klug, 1824) analyzed in the study. ∑TL: total length; KL mean karyotype length (= ∑TL/2n) ± SD: standard deviation; CV coefficient of variation (= ± SD/KL); Video S1: *Cephalotes pusillus* (Klug, 1824) digging behavior. The movie shows workers actively unloading the wood fragments from inward colony.
