# Supplementary material for: Reassessing the Chromosome Number and Morphology of the Turtle Ant Cephalotes pusillus (Klug, 1824) Using Karyomorphometrical Analysis and Observations of New Nesting Behavior
Source: Insects. 2017 Oct 23;8(4):114. doi: 10.3390/insects8040114 (PMC5746797; doi:10.3390/insects8040114)
Supplement: Supplementary file 1 [file insects-08-00114-s001.zip › insects-228094-supplementary/insects-228094-Supplement Table S1.docx]

**Table S1**. Karyomorphometrical analyses of the eight specimens of *Cephalotes pusillus* (Klug, 1824) analyzed in the study. ∑TL: total length; KL mean karyotype length (= ∑TL/2n) ± SD: standard deviation; CV coefficient of variation (= ± SD/KL). All measurements are given in “μM”.

| Chromosome | Specimen | | | | | | | |
| --- | --- | --- | --- | --- | --- | --- | --- | --- |
|  | **1** | **2** | **3** | **4** | **5** | **6** | **7** | **8** |
| 1 | 3.91 | 2.72 | 3.05 | 3.03 | 4.44 | 3.22 | 4.48 | 2.98 |
| 1 | 3.49 | 2.37 | 2.93 | 2.71 | 4.40 | 3.14 | 4.26 | 2.79 |
| 2 | 2.81 | 2.18 | 2.13 | 2.14 | 3.16 | 2.52 | 3.26 | 2.74 |
| 2 | 2.54 | 2.09 | 2.10 | 2.12 | 3.11 | 2.48 | 3.12 | 2.74 |
| 3 | 2.51 | 1.90 | 1.90 | 2.07 | 2.88 | 2.38 | 2.88 | 2.69 |
| 3 | 2.38 | 1.88 | 1.87 | 1.99 | 2.77 | 2.23 | 2.84 | 2.42 |
| 4 | 2.34 | 1.82 | 1.87 | 1.99 | 2.60 | 2.22 | 2.82 | 2.37 |
| 4 | 2.32 | 1.75 | 1.84 | 1.97 | 2.51 | 2.13 | 2.74 | 2.24 |
| 5 | 2.24 | 1.75 | 1.75 | 1.96 | 2.49 | 2.11 | 2.63 | 2.24 |
| 5 | 2.16 | 1.74 | 1.73 | 1.84 | 2.35 | 2.09 | 2.61 | 2.23 |
| 6 | 2.03 | 1.67 | 1.73 | 1.82 | 2.32 | 2.03 | 2.57 | 2.18 |
| 6 | 2.01 | 1.61 | 1.69 | 1.81 | 2.28 | 2.03 | 2.57 | 2.17 |
| 7 | 1.99 | 1.60 | 1.62 | 1.79 | 2.28 | 2.01 | 2.51 | 2.11 |
| 7 | 1.96 | 1.47 | 1.62 | 1.71 | 2.22 | 1.83 | 2.50 | 2.02 |
| 8 | 1.93 | 1.40 | 1.55 | 1.68 | 2.15 | 1.75 | 2.38 | 1.97 |
| 8 | 1.86 | 1.33 | 1.50 | 1.65 | 2.11 | 1.69 | 2.34 | 1.90 |
| 9 | 3.35 | 2.87 | 2.81 | 3.06 | 3.80 | 3.10 | 3.97 | 3.21 |
| 9 | 3.13 | 2.53 | 2.51 | 3.05 | 3.67 | 2.79 | 3.95 | 3.10 |
| 10 | 2.81 | 2.44 | 2.44 | 2.44 | 3.39 | 2.77 | 3.36 | 2.77 |
| 10 | 2.57 | 2.28 | 2.37 | 2.40 | 3.29 | 2.37 | 3.32 | 2.68 |
| 11 | 2.50 | 2.18 | 2.26 | 2.36 | 3.10 | 2.31 | 2.91 | 2.68 |
| 11 | 2.48 | 2.17 | 2.21 | 2.26 | 2.87 | 2.23 | 2.91 | 2.64 |
| 12 | 2.37 | 2.10 | 2.10 | 2.23 | 2.86 | 2.20 | 2.81 | 2.52 |
| 12 | 2.36 | 2.08 | 2.09 | 2.12 | 2.77 | 2.16 | 2.79 | 2.49 |
| 13 | 2.32 | 2.03 | 2.04 | 2.04 | 2.74 | 2.13 | 2.78 | 2.38 |
| 13 | 2.30 | 1.92 | 2.03 | 2.03 | 2.66 | 2.09 | 2.78 | 2.37 |
| 14 | 2.26 | 1.84 | 1.97 | 2.01 | 2.62 | 2.09 | 2.74 | 2.18 |
| 14 | 2.10 | 1.82 | 1.89 | 2.00 | 2.58 | 2.08 | 2.74 | 2.16 |
| 15 | 1.96 | 1.78 | 1.87 | 1.94 | 2.58 | 2.04 | 2.73 | 2.11 |
| 15 | 1.90 | 1.76 | 1.82 | 1.93 | 2.52 | 2.03 | 2.65 | 2.09 |
| 16 | 1.83 | 1.74 | 1.80 | 1.92 | 2.50 | 2.02 | 2.62 | 2.02 |
| 16 | 1.76 | 1.70 | 1.78 | 1.90 | 2.48 | 1.96 | 2.56 | 1.93 |
| 17 | 1.75 | 1.68 | 1.77 | 1.83 | 2.45 | 1.90 | 2.25 | 1.92 |
| 17 | 1.52 | 1.68 | 1.62 | 1.72 | 2.32 | 1.68 | 2.23 | 1.70 |
| 18 | 2.54 | 1.84 | 2.08 | 2.23 | 2.78 | 2.34 | 3.00 | 2.56 |
| 18 | 2.42 | 1.82 | 2.06 | 2.06 | 2.76 | 2.22 | 2.88 | 2.43 |
| 19 | 2.39 | 1.81 | 1.89 | 2.06 | 2.66 | 2.10 | 2.63 | 2.37 |
| 19 | 2.32 | 1.81 | 1.83 | 2.03 | 2.59 | 2.03 | 2.61 | 2.18 |
| 20 | 2.28 | 1.80 | 1.82 | 1.99 | 2.38 | 1.98 | 2.61 | 2.16 |
| 20 | 2.21 | 1.74 | 1.77 | 1.92 | 2.37 | 1.98 | 2.59 | 2.14 |
| 21 | 2.15 | 1.60 | 1.65 | 1.77 | 2.25 | 1.95 | 2.59 | 2.10 |
| 21 | 1.99 | 1.56 | 1.61 | 1.76 | 2.20 | 1.91 | 2.55 | 2.02 |
| 22 | 1.86 | 1.48 | 1.49 | 1.75 | 2.08 | 1.91 | 2.54 | 2.02 |
| 22 | 1.79 | 1.42 | 1.41 | 1.72 | 2.08 | 1.76 | 2.42 | 1.60 |
|  |  |  |  |  |  |  |  |  |
| ∑TL | 101.72 | 82.75 | 85.87 | 90.82 | 119.43 | 96.00 | 125.03 | 102.31 |
| KL | 2.31 | 1.88 | 1.95 | 2.06 | 2.71 | 2.18 | 2.84 | 2.33 |
| ±SD | 0.47 | 0.34 | 0.37 | 0.35 | 0.55 | 0.36 | 0.49 | 0.36 |
| CV | 0.21 | 0.18 | 0.19 | 0.17 | 0.20 | 0.16 | 0.17 | 0.15 |
